# Dynamics of the Drosophila Circadian Clock: Theoretical Anti-Jitter Network and Controlled Chaos

**DOI:** 10.1371/journal.pone.0011207

**Published:** 2010-10-13

**Authors:** Hassan M. Fathallah-Shaykh

**Affiliations:** Departments of Neurology, Mathematics, Cell Biology, and Biomedical and Mechanical Engineering, The University of Alabama at Birmingham and the UAB Comprehensive Neuroscience and Cancer Centers, Birmingham, Alabama, United States of America; Tel Aviv University, Israel

## Abstract

**Background:**

Electronic clocks exhibit undesirable jitter or time variations in periodic signals. The circadian clocks of humans, some animals, and plants consist of oscillating molecular networks with peak-to-peak time of approximately 24 hours. Clockwork orange (CWO) is a transcriptional repressor of Drosophila direct target genes.

**Methodology/Principal Findings:**

Theory and data from a model of the Drosophila circadian clock support the idea that CWO controls anti-jitter negative circuits that stabilize peak-to-peak time in light-dark cycles (LD). The orbit is confined to chaotic attractors in both LD and dark cycles and is almost periodic in LD; furthermore, CWO diminishes the Euclidean dimension of the chaotic attractor in LD. Light resets the clock each day by restricting each molecular peak to the proximity of a prescribed time.

**Conclusions/Significance:**

The theoretical results suggest that chaos plays a central role in the dynamics of the Drosophila circadian clock and that a single molecule, CWO, may sense jitter and repress it by its negative loops.

## Introduction

**Figure 1:**
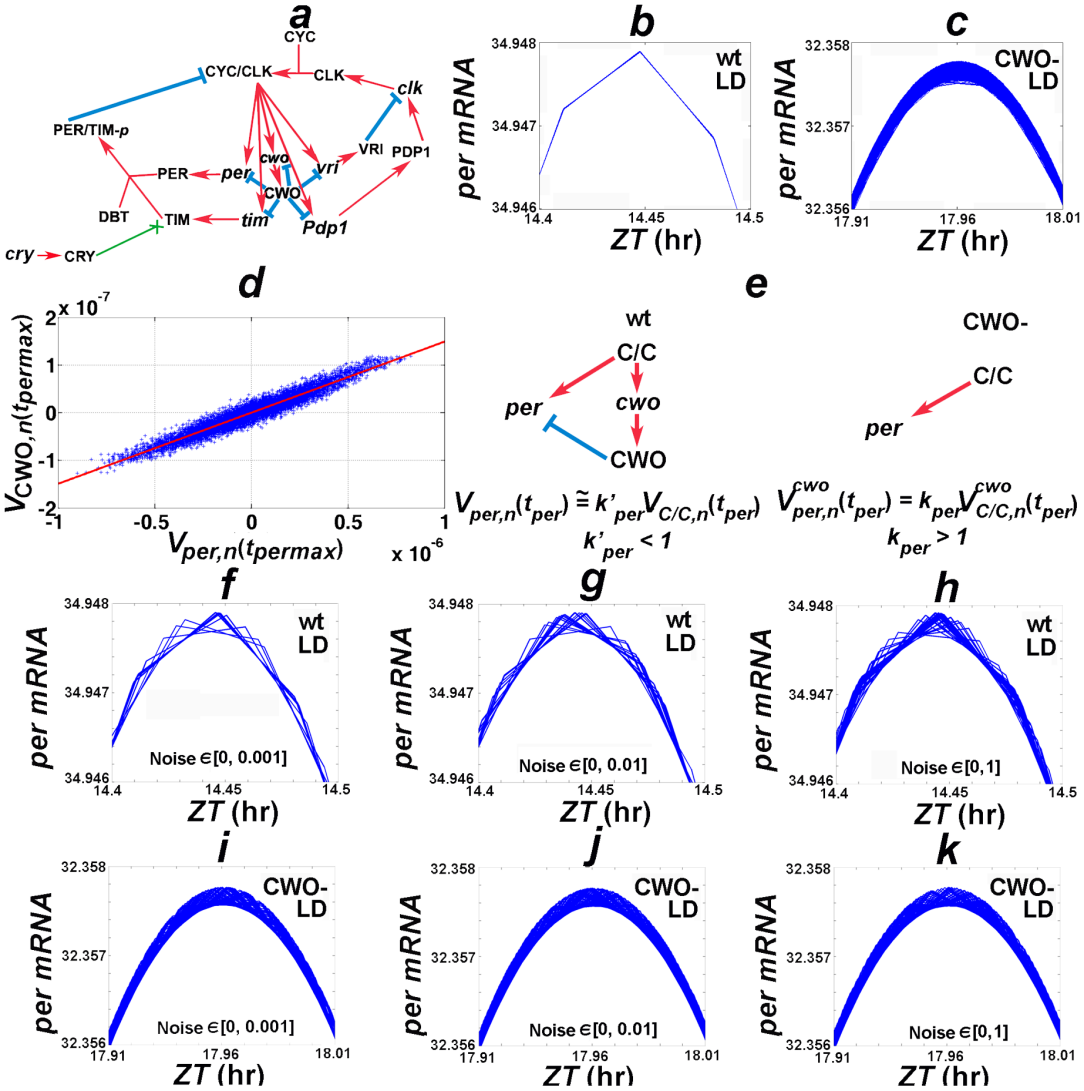
Network model and jitter. (*a*) is a cartoon depicting the *Drosophila* circadian molecular network; protein and mRNA are represented by capital letters and lower case, respectively. Red arrows and cyan blocked lines indicate stimulatory and inhibitory interactions, respectively. The green arrow ending in X indicates that CRY protein enhances the degradation of TIM. (*b*) and (*c*) plot recurring orbits of the wt and *cwo*-mutant models in LD (cycles 100 to 120000), respectively; observe the jitter/variation when CWO is absent. The variability of CWO is proportional to the variability of each direct target gene at the times of its peak and trough (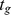, see File S1):
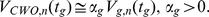
(*d*) plots the variability of CWO (y-axis) vs. *per* mRNA (*x*-axis) at the peak-time of *per*. (*e*) illustrates the theoretical results predicting that the jitter of CWO dampens the jitter of direct targets at the times of their peaks and troughs. (*f*)–(*k*) plot *per* oscillations in simulations where the CLK/CYC of each cycle (total = 70) of the wt (*f–h*) and the *cwo*-mutant (*i–k*) models is pulsed at ZT = 14 hr by pseudorandom numbers drawn from a uniform distribution on [0, 0.001] (*f* and *i*), [0, 0.01] (*g* and *j*), and the unit interval (*h* and *k*), respectively. The unit of the y-axes of *b–c* and *f–k* is arbitrary.

Humans, most animals and plants, have a biological clock that exhibits circadian rhythms that control the timing of sleep, alertness, and appetite. Circadian clocks exhibit 24-hr recurring behavioral and transcriptional oscillations, generated by interconnected transcriptional feedback loops (see File S1). In particular, the Drosophila circadian clock has one positive and two negative loops that interconnect at CLK-CYC, a heterodimer of the CLOCK (CLK) and CYCLE (CYC) proteins. CLK-CYC binds canonical E-box sequences to activate the transcription of direct targets clockwork orange (*cwo*), period (*per*), timeless (*tim*), vrille (*vri*), and par domain protein 1 (*Pdp1*, Figure 1a) [1]–[6]. CWO is a recently defined negative transcriptional regulator of the same direct targets as those of CLK-CYC (Figure 1a). The presence of circadianly expressed *cwo*-orthologs in mouse (*dec1* and *dec2*), suggest that a similar feedback mechanism exists in mammals [7], [8]; this view may also extend to other animal systems [9].

A recent report describes a mathematical model of the Drosophila circadian clock. This model is faithful in the sense that it replicates biological results (see File S1 and [10]). In particular, simulations generate timely oscillations with peak-to-peak times approximately 24 hours in LD and DD, and entrainment in response to light shifts; furthermore, simulations replicate biological data from flies with *cwo*-, *clk*-, and dPDBD-mutations as well as from experiments that enhance the activity of CLK/CYC.

Typically, electronic clocks exhibit jitter or undesirable variations in periodic signals. Interestingly, like digital clocks and unlike the wt model, the *cwo*-mutant model of the Drosophila circadian clock exhibits jitter or variations in recurring signal (see Figures 1b–c). Here, I investigate the idea that CWO regulates an anti-jitter control system and study its contribution to the dynamics of the circadian model.

## Results

### Theory

Zeitgeber time (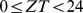) refers to time *modulo* 24 where 0–12 hr and 12–24 hr indicate light and dark cycles (LD), respectively. The cycle-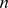 variability in the concentration (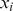) of a molecule, 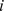, at 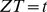 is computed as:
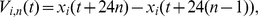
which computes the difference in concentration between cycles 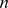 and 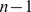. The *cwo*-mutant model predicts that the variability in the concentration of each direct target gene, 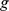, at the times of its peak or trough, 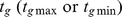, is proportional to the variability of CLK-CYC (C/C, see File S1); in particular,
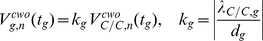
(1)The parameters 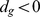 and 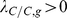 are the decay rates of direct target mRNAs and the regulatory weights that encode the CLK-CYC-mediated transcriptional activation, respectively. The derivation of this equation uses the fact that the relationship between the molecules that regulate 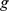 is linear at its peak and trough (see File S1). Notice that 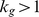 for *per* and *tim*.

In the case of the wt model, the peak-time linear relationships between the variability of CWO and the variability of each direct target gene (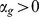, see Figure 1d) also lead to:
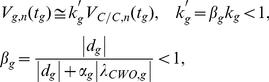
(2)where 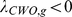 are regulatory weights that encode the CWO-mediated repressive actions (see Figure 1 and File S1). Interestingly, 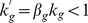 for all direct targets. Equations (1) and (2) reveal that the cycle-to-cycle peak-time variability of *per* and *tim* in the *cwo*-mutant model is always larger than the variability of CLK/CYC. CWO seems to lower this cycle-to-cycle jitter because its own variability, proportional to each direct target gene, is subtracted by its negative repressive actions. Notice that the design of the CWO negative control circuit is similar to the idea of digital phase-locked negative loops in the sense that the variability of CWO, proportional to the variability of each direct target, is fed back by the negative loops to dampen the variability of each direct target (see Equations 1–2 and Figure 1e). Therefore, Equations (1) and (2) predict that CWO lowers the cycle-to-cycle variability in each direct target gene at least at the times of its peak and trough.

### Biological systems can be noisy

To study how the wt and *cwo*-mutant networks react to errors, CLK/CYC is pulsed with noise at ZT = 14 hours, i.e. near its peak. The data reveal that the wt model shows less *per* variability/jitter than the *cwo*-mutant model even when noise is drawn from the unit interval (Figures 1f–1k). These findings validate the theoretical results.

The following quantities 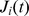 and 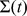 are called, respectively, the jitter of molecule, 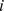, and of the circadian network at 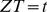:
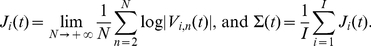
(3)

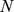
 and 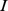 refer to the total number of cycles and oscillating molecules, respectively. The circadian jitter is the mean of the molecular jitters.

### CWO is an anti-jitter molecule in LD

**Figure 2:**
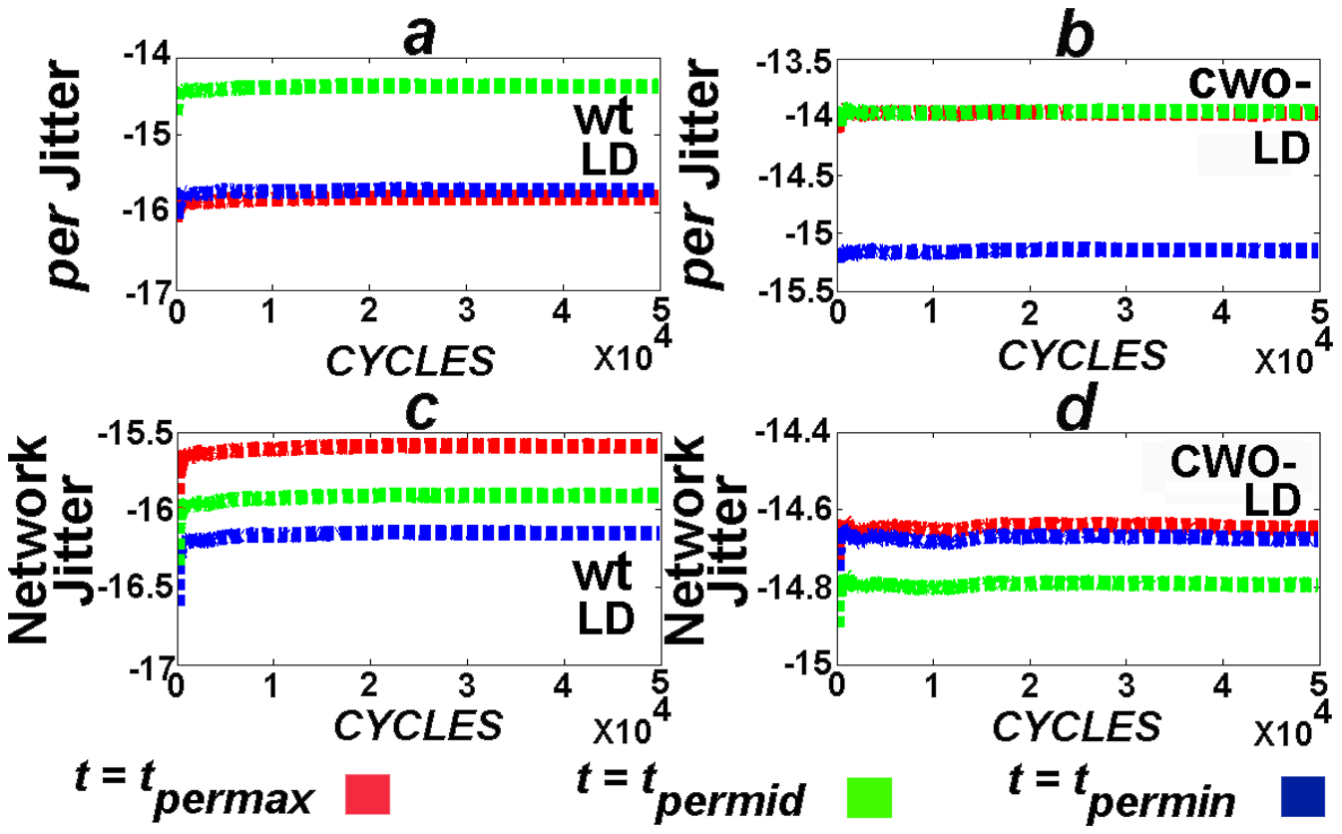
CWO dampens molecule and network jitters in LD. To avoid the complications of numerical integration over long periods, the integration of these experiments is performed from 0 to 24 hr while computing measurements only at two fixed time points, 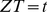 and 24 hr; 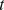 corresponds to either the time of the peak (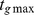), trough (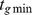) of each direct target gene or 
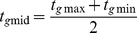
. The procedure is then repeated with the last vector of the previous cycle as initial condition. The numerical integration methods are based on an explicit Runge-Kutta formula, the Dormand-Prince pair (ode45, Matlab), and on a variable order solver based on the numerical differentiation formulas (ode15s, Matlab). Relative error tolerance is 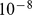. Data from ode45 are shown here, the results from ode15s are shown in Figure S4. (*a*) and (*b*) plot the jitter of *per* at 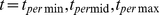 in the wt and *cwo*-mutant models in LD, respectively (see Equation 3). (*c*) and (*d*) plot the network jitter of the wt and *cwo*-mutant models in LD, respectively. Notice that the limits converge and that *per* and network jitters are larger in the *cwo*-mutant model as compared to wt. Similarly, *tim*, *cwo*, *pdp1* and *vri* jitters are also larger in the *cwo*-mutant models as compared to wt (Figures S1, S2, and S3). Network jitter is lower in the presence of CWO at 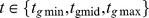, where 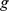 refers to direct target genes. These times include *ZT* = 2.91, 4.19, 4.22, 5.2, 5.22, 8.68, 10.07, 10.15, 10.8, 10.99, 14.44, 15.95, 16.4, 16.77 and 19.09 in the wt model and *ZT* = 6.42, 7.58, 7.88, 7.94, 8.98, 12.19, 13.31, 13.75, 13.76, 14.97, 17.96, 19.04, 19.623, 19.57 and 20.97 in the *cwo*-mutant model (see Figures S1, S2, and S3).

The goals of the following computations are to evaluate the theoretical results and study the effects of CWO on the jitter of each direct target gene and of the entire network. The analysis is done in LD conditions; the system is integrated numerically from 0 to 24 hrs and data is collected only at a prescribed time (Figure 2). As predicted, the results reveal that CWO actions dampen jitter of the whole network as well as the jitter of direct target genes not only at the times of their peaks and troughs. In particular, the wt network jitter is lower than the *cwo*-mutant model at 15 independent times that span both light and dark cycles (Figures 2, S1, S2, and S3). The fact that two independent integration methods (ode45 and ode15s) yield consistent results enhances my confidence in these findings (Figure S4).

### Stable limit cycles and stable phase in LD

**Figure 3:**
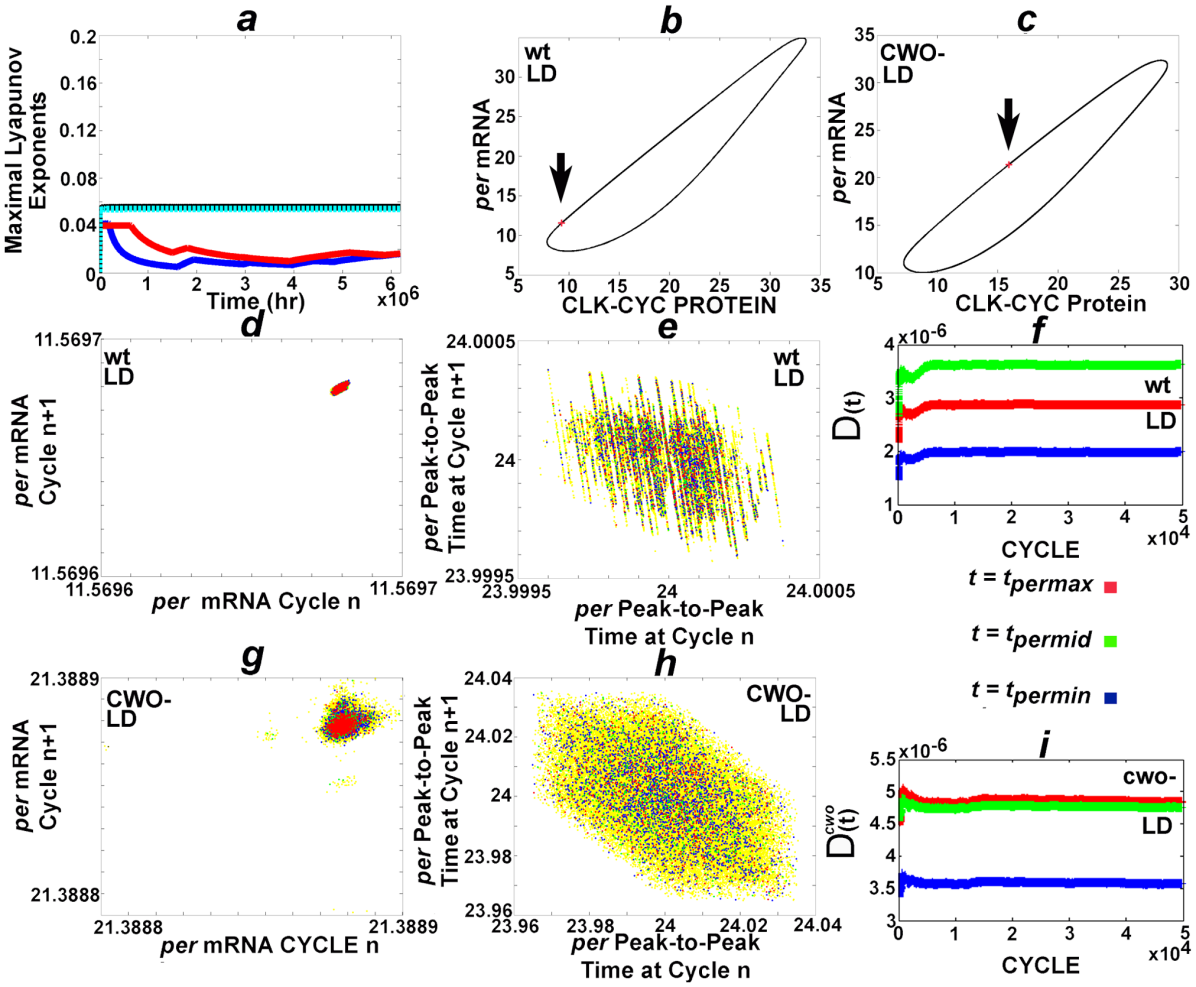
Chaotic attractors and almost periodic orbits in LD. (*a*) plots the positive maximal Lyapunov characteristic exponents of the wt model in LD (blue), wt model in DD (black), *cwo*-mutant model in LD (red), and the *cwo*-mutant model in DD (cyan), respectively. (*b*) and (*c*) plot the orbits from cycle 100 to 120,000 of the wt and *cwo*-mutant models in LD, respectively. Observe that the trajectories remain confined to limit cycles. In addition, the orbits revisit the same neighborhoods at *ZT* = 0 in LD (arrows pointing to red X). (*d*) and (*g*) are recurrence plots of consecutive levels of *per* (arbitrary unit) at *ZT* = 0 in the wt and *cwo*-mutant models, respectively; the following cycles are plotted in this order, 1) cycles 70,000 to 120,000 (yellow), 2) 115,000–120,000 (blue), 3) 115,00–116,000 (green), and 4)119,000–120,000 (red). (*e*) and (*h*) are recurrence plots of consecutive peak-to-peak times (hr) of *per* in the wt and *cwo*-mutant models, respectively; the following cycles are plotted in this order, 1) cycles 70,000 to 120,000 (yellow), 2) 115,000–120,000 (blue), 3) 115,00–116,000 (green), and 4)119,000–120,000 (red). (*f*) and (*i*) plot 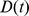 and 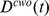 for 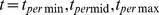; similarly, 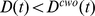 for 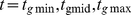, where 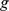 refers to direct target genes *tim*, *cwo*, *pdp1*, and *vri*. The unit of the y-axes of (*b*) and (*c*) is arbitrary.

Typically, chaotic systems exhibit dynamics that are highly sensitive to initial conditions resulting in exponential growth of small perturbations in the initial conditions. The Lyapunov exponents (LE) describe the stability of nonlinear systems by measuring the exponential divergence or convergence of infinitesimally close trajectories. A positive LE is taken as an indication that the system is chaotic. I apply the discrete QR method with orthonormalization at each step to compute the LE (see File S1) [11]–[13]. The findings reveal that the maximal LE converge to positive real numbers thus providing evidence for chaotic dynamics of the wt and *cwo*-mutant models in both LD and DD conditions (Figures 3a and S5). The wt model has two positive LE in both LD and DD; the cwo-mutant model has 2 and a single positive LE in LD and DD conditions, respectively (Figure S5). The LE were computed over 6.1×10^6^ hours. The averages of the maximal LE of the last million hours are 0.0141, 0.016, 0.0555, and 0.0539 in the wt LD, *cwo*-mutant LD, wt DD, and *cwo*-mutant DD models, respectively; the standard deviations are 0.0012, 4.5436×10^−4^, 1.3224×10^−6^, and 4.8050×10^−7^, respectively. The averages and standard deviations of the second positive LE of the wt LD, *cwo*-mutant LD, and wt DD models are, respectively, [1.6782×10^−4^,1.5719×10^−5^], [1.3513×10^−6^, 3.8408×10^−6^], and [7.8549×10^−4^, 4.3632×10^−7^].

The algorithm for computing the LE of the clock models is applied to the classical Lorenz attractor. The LE of the Lorenz attractor converge at 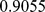, 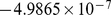, and 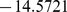, which are in agreement with the literature (see File S1 and Figure S5b). These findings enhance my confidence in the computation of the LE.

Phase-space graphs in LD conditions reveal trajectories of the wt and *cwo*-mutant models that are attracted to stable limit cycles (Figures 3b–c, S6, S7, S8, and S9), which are consistent with chaotic attractors in the sense that the orbits are confined to small subsets of the space. Furthermore, the position of *ZT* = 0 remains restricted to a very small neighborhoods on the limit cycles of the wt and *cwo*-mutant models in LD (see Figures 3b–c arrows).

### CWO stabilizes recurrence time and phase in LD

To plot recurrence maps, the state of the network at 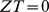 is recorded as days (cycles) advance. Here, the numerical integration method (ode45) uses variable time steps from 0 to 24 hr; this procedure is repeated with the last vector of the previous cycle as initial condition. To ensure that the results are not biased by the discretization procedure, the integration is also performed with smaller maximal time steps (×½ and ×1/8). The recurrence maps consistently reveal that, as compared to wt, the molecular levels of the *cwo*-mutant model exhibit larger variability at *ZT* = 0 (Figures 3d, 3g and S10). Dynamical systems are periodic in the mathematical sense if they revisit the same points or exact values. Since the models are not periodic (Figure 3), I will use the term peak-to-peak time instead of period. The orbits of the wt and *cwo*-mutant models are *almost* periodic in the sense that each orbit revisits a very small neighborhood of the phase space at the end of each LD cycle (Figures 3b–c, 3d, and 3g). Because a periodic multidimensional biological network may be excessive as it requires significant control, an almost periodic orbit seems like a practical solution.

Previous results showed that peak-to-peak time is inversely proportional to *per* mRNA levels within bounds (see [10]). Furthermore, *per* mRNA levels exhibit larger variability in the absence of CWO (Figures 3d and 3g). Thus, it is not surprising that the absence of CWO leads to larger variability in peak-to-peak times (see Figures 3e and 3h). Specifically, peak-to-peak times vary within 24 hr±1.8 seconds and 24 hr±2.4 minutes in the wt and *cwo*-mutant models, respectively.

Figures 2 and 3 reveal that the actions of CWO dampen jitter and suggest that CWO decreases the size of the small neighborhood revisited by the trajectory at any fixed *ZT*. To estimate the dimension of this neighborhood, I examine the quantity D, which reflects the average Euclidean distance of the recurring orbit from a single point within the attractor at 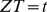,
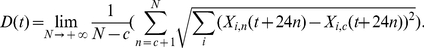
(4)Here *c* refers to a cycle number such that the forward orbit remains confined to the chaotic attractor/limit cycle; *c* is taken as 100. The findings reveal that the neighborhood revisited by the orbit at a fixed *ZT* is confined to a sphere and CWO reduces the radius of this sphere in LD (Figures 3f and 3i).

### Phase shifts in DD

**Figure 4:**
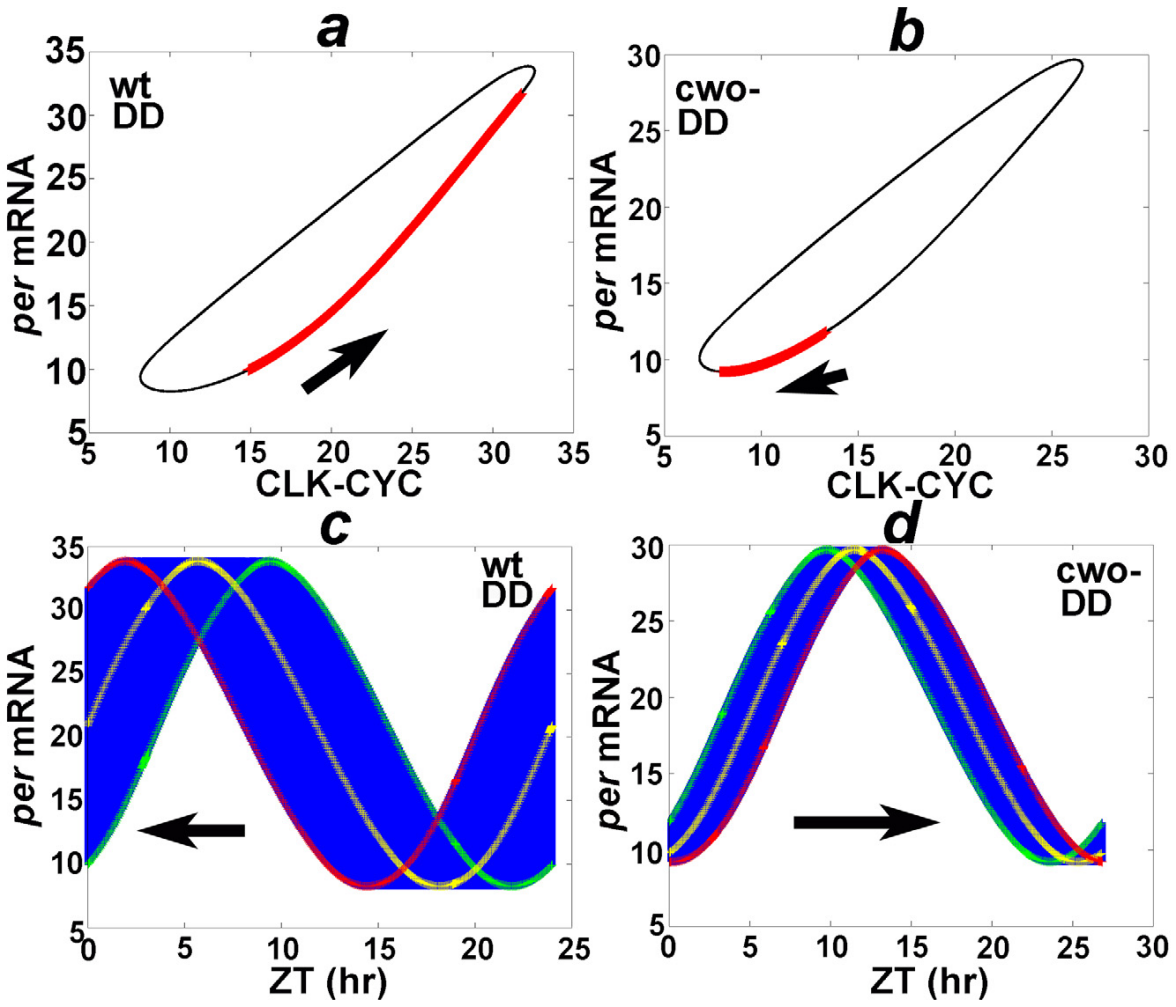
Phase shifts in DD. (*a*) and (*b*) plot the trajectories of the orbit from cycle 100 to 120,000 of the wt and *cwo*-mutant models in DD, respectively. Observe that the trajectories remain confined to limit cycles. However, unlike the limit cycles in LD (Figures 3b–c), the position of *ZT* = 0 (red X) in the limit cycle migrates in the direction of the arrows as cycles advance (*a*, cycles 100 to 20,000; *b*, cycles 100 to 120000). (*c*) and (*d*) plot recurring orbits of the wt and *cwo*-mutant models in DD; the phase exhibits minute shifts to the left and to the right at the end of each cycle in the wt and *cwo*-mutant models, respectively (arrows). The *per* mRNA oscillation in the first (cycle = 100), middle (*c*, cycle = 10000; *d*, cycle = 60000) and last cycles (*c*, cycle = 20000; *d*, cycle = 120000) are labeled in green, yellow, and red respectively. The unit of the y-axis is arbitrary.

Like LD cycles, phase-space graphs in DD conditions also reveal trajectories that converge to stable limit cycles/chaotic attractors (Figures 4a–b, S7, and S9). However, unlike the results in LD, the phase exhibits minute shifts to the left and right after each DD cycle in the wt and *cwo*-mutant models, respectively (Figure 4, arrows). These findings highlight the critical importance of light in resetting the phase of the clock each day by confining each molecular peak to the proximity of a prescribed time (see Figure 3).

## Discussion

The theory and results detailed in this paper support the conclusion that CWO appears to control negative circuits that reduce jitter in the Drosophila circadian clock leading to stabilization of peak-to-peak time. There is no current data from *cwo*-mutant flies that is relevant to the dynamics of the clock. Nonetheless, experiments could be designed to validate these predictions; like a detailed analysis of variability in peak-to peak times between wt and *cwo*-mutant flies. This is the first example of a putative molecular anti-jitter negative circuit; it is remarkable that designs that reduce jitter in electronic clocks are similar to the negative circuits controlled by CWO.

The theoretical results reveal that peak-to-peak times vary within 24 hr±1.8 seconds and 24 hr±2.4 minutes in the wt and *cwo*-mutant models, respectively. This translates to an 80-fold difference generated by a jitter that appears after the 3^rd^ place after the decimal point; a peak-to-peak time of 24 hours is equal to 1440 minutes or 86,400 seconds. Chaotic attractors have been described in dynamical biological systems like the heart rate, cell division, oscillatory enzymatic reactions, and calcium oscillations [14], [15]. Prior to the discovery of *cwo*, Tsumoto et al. and Leloup et al. reported phase-space graphs from a model of the Drosophila circadian clock consistent with either chaos or birhythmicity [16], [17]. The positive maximal LE, reported here, demonstrate that both the wt and *cwo*-mutant models of the Drosophila circadian clock are chaotic in LD and DD. Nevertheless, the orbits are confined to limit cycles supporting the idea of chaotic attractors. Daily light appears to play a critical role in resetting the phase by limiting the molecular peaks to prescribed times.

## Methods

Simulations are performed in Matlab (Mathworks, Natick, MA) at the Dense Memory Cluster of the Alabama Supercomputer Center (www.asc.edu). Details of the model, theory, and computations are included in File S1.

## Supporting Information

File S1 Supplementary material.
(0.41 MB DOC)

Figure S1 CWO lowers *tim* and network jitters. Shown are the *tim* and network jitters of the wt (*a,c*) and *cwo*-mutant (*b,d*) models in LD at t ∈ {t*_timmin_*, t*_timmid_*, t*_timmax_*} starting from cycle 100.
(2.37 MB TIF)

Figure S2 CWO lowers *Pdp1* and network jitters. Shown are the *Pdp1* and network jitters of the wt (*a,c*) and *cwo*-mutant (*b,d*) models in LD at *t *∈ {*t_pdpmin_*, *t_pdpmid_*, *t_pdpmax_*} starting from cycle 100.
(2.96 MB TIF)

Figure S3 CWO lowers *vri* and network jitters. Shown are the *vri* and network jitters of the wt (*a,c*) and *cwo*-mutant (*b,d*) models in LD at *t *∈ {*t_vrimin_*, *t_vrimid_*, *t_vrimax_*} starting from cycle 100.
(2.52 MB DOC)

Figure S4 CWO lowers direct target and network jitters, second numerical method. These results are computed by ode15s (see Figure 2 legend); shown are the plots of *per* and network jitters of the wt (*a,c*) and *cwo*-mutant (*b,d*) models in LD at *t *∈ {*t_permin_*, *t_permid_*, *t_permax_*} starting from cycle 100.
(3.19 MB TIF)

Figure S5 The Lyapunov characteristic exponents. (*a*) plots the second positive LE of the wt model in LD (blue), wt model in DD (black) and the *cwo*-mutant model in LD (red). (*b*) plots the LE for the Lorenz equations (σ = 10, ρ = 28 and β = 8/3). (*c–f*) plot the full LE spectrum of the wt and *cwo*-mutant models in LD and DD conditions.
(2.87 MB TIF)

Figure S6 Attractor to stable limit cycle; wt model in LD. Shown are the trajectories of the wt model in LD starting from different points in the phase space (red X) and converging to a stable limit cycle (cycles 1–120000).
(2.38 MB TIF)

Figure S7 Attractor to stable limit cycle; wt model in DD. Shown are the trajectories of the wt model in DD starting from different points in the phase space (red X) and converging to a stable limit cycle (cycles 1–120000).
(2.38 MB TIF)

Figure S8 Attractor to stable limit cycle; *cwo*-mutant model in LD. Shown are the trajectories of the *cwo*-mutant model in LD starting from different points in the phase space (red X) and converging to a stable limit cycle (cycles 1–120000).
(2.33 MB TIF)

Figure S9 Attractor to stable limit cycle, *cwo*-mutant model in DD. Shown are the trajectories of the *cwo*-mutant model in DD starting from different points in the phase space (red X) and converging to a stable limit cycle (cycles 1–120000).
(2.33 MB TIF)

Figure S10 Jitters persist after lowering maximal time steps. Shown are the variations in *per* mRNA oscillations of the wt (*a–c*) and *cwo*-mutant (*d–f*) models (cycles 100–1500) when the maximal time step of ode45 is not changed (*a* and *d*), multiplied by 1/2 (*b* and *e*) and 1/8 (*c* and *f*).
(2.70 MB TIF)
