# Supplementary material for: Dynamics of the Drosophila Circadian Clock: Theoretical Anti-Jitter Network and Controlled Chaos
Source: PLoS One. 2010 Oct 13;5(10):e11207. doi: 10.1371/journal.pone.0011207 (PMC2954144; doi:10.1371/journal.pone.0011207)
Supplement: File S1 — Supplementary material. (0.41 MB DOC) [file pone.0011207.s001.doc]

**SUPPLEMENTARY INFORMATION**

**Dynamics Of The Drosophila Circadian Clock: Theoretical Anti-Jitter Network And Controlled Chaos**

Hassan M Fathallah-Shaykh

The University of Alabama at Birmingham, Departments of Neurology, Mathematics, Cell Biology, and Biomedical and Mechanical Engineering, and the UAB Comprehensive Neuroscience and Cancer Centers, Birmingham, AL, USA.

**The Molecular Network**

Glossop *et* *al.* described two negative interlocked feedback loops within the Drosophila circadian oscillator: 1) a per/tim loop, which is activated by CLK-CYC and repressed by the PER-TIM dimer, and 2) the vri/clk loop consisting of the CLK-CYC heterodimer activating VRI, which represses *clk* transcription [1–4]. The Pdp1/clk positive loop, which also interconnects at CLK-CYC, includes PDP1 acting as a transcriptional activator of *clk* mRNA (Figure 1a) [5–7]. PER-TIM represses the transcriptional ability of CLK-CYC by inhibiting its DNA binding activity [8–11]; furthermore, Double-Time (DBT) kinase appears to mediate these effects on CLK-CYC by phosphorylating PER and CLK [12–14]. DBT is incorporated in our model as a positive and necessary regulator of the PER-TIM dimer. CRY is a light-regulated cryptochrome that leads TIM to its subsequent degradation [15].

**System of Ordinary Differential Equations**

The system of ODE was introduced elsewhere [15]. Assuming that genes/proteins $j\in\left\{1,...,n\right\}$ regulate the production of gene/protein $i$, I use the following type of differential equations as a general model;

$\begin{matrix} \frac{dx_{i}(t)}{dt}=\rho_{i}g(u_{i})x_{i}(t)(s_{i}-x_{i}(t)),\text{ }1\leq j\leq n, \\ u_{i}=\sum_{j=1}^{n} \lambda_{ji}x_{j}(t)-d_{i}x_{i}(t), \\ g(u_{i})=\frac{u_{i}}{\sqrt{1+u_{i}^{2}}}. \end{matrix}$, **(1)**

where $x_{i}$ is the state vector representing the concentration of molecule $i$ at its site of action. The real parameters $\lambda_{ji}$ are regulatory weights that encode the effects of molecule $j$ on the production rate of molecule $i$. Positive and negative $\lambda_{ji}$ are interpreted as $j$ activates or represses $i$, respectively. The absolute value of $\lambda_{ji}$ reflects the strength of stimulation or repression. The sum of the regulatory influences is modulated by an odd sigmoid function, of the form:

$g(u)=\frac{u}{\sqrt{1+u^{2}}}=\tanh(\ln(u+\sqrt{1+u^{2}}))$,

together with a real parameter $\rho_{i}>0,$ indicating the maximal rate of formation of $i$. The term $u_{i}$ reflects the sum of the regulatory forces acting on molecule $i$. The model incorporates logistic terms [$(x_{i})(s_{i}-x_{i})$], which include constants $s_{i}\geq0,$ indicating the saturation level of molecule $i.$ The real parameter $d_{i}$ is the decay rate of $i$.

**Summary of Previous Results**

The system of equations in (1) is nonlinear; nonetheless, notice that because the oscillations do not reach the saturation level (max) or 0 (min),

$$\frac{dx_{i}}{dt}=0\Leftrightarrow u_{i}=0.$$

This result implies that the relationship between the molecules that regulate molecule $i$ is linear at the peaks and troughs. This linearity at the peaks and troughs is a key feature of this system of equations that will be used in developing the theory below.

The parameters were chosen such that the system of ODE generates indefinite oscillations with timely peaks of the mRNAs and proteins. Furthermore, the model is robust because parameter perturbations replicate biological phenotypes of the clock that were not used in fitting the parameters. These phenotypes include: 1) entrainment in response to day/light shifts, 2) the states of the clock when *clk*, *cyc*, and dPDBD are mutated, 3) period changes when the activity of CLK/CYC is modulated, 4) the paradoxical effects of *cwo* mutations on the peaks, 5) a peak-to-peak time of 26.8 hr in *cwo*-mutants in DD conditions (see [15]).

**New Theory**

I use the symbol $g$ to refer to the direct target genes *per*, *tim*, *vri*, *pdp1*, and *cwo*. Let $t_{g}$and $t_{g}^{cwo}$refer to the peak or trough times of $g$in the wt and *cwo*-mutant models in LD, respectively. Let $x_{i,n}(t)$ and $x_{i,n}^{\text{cwo}}(t)$ denote the concentration levels of a molecule, $i,$ at time = $t$ of cycle $n$ in the wt and mutant models, respectively. Define

$x_{i,n}(t)=x_{i}(t+24n)$and $x_{i,n}^{cwo}(t)=x_{i}^{cwo}(t+24n)$ **(2.0)**

Also define $V_{i,n}(t_{g}),$ the variation of $i$ in the cycle $n$ as,

$V_{i,n}(t_{g})=x_{i,n}(t)-x_{i,n-1}(t)=x_{i}(t_{g}+24n)-x_{i}(t_{g}+24(n-1));$ **(2.1)**

The symbol $C/C$ refers to CLK-CYC. The system of ordinary differential equations (Equation 1) shows that at the peaks (maxima) and troughs (minima) of$g$in cycle $n,$

$$\lambda_{C/C,g}x_{C/C,n}^{\text{cwo}}(t_{g})-\left|d_{g}\right|x_{g,n}^{\text{cwo}}(t_{g}^{cwo})=0\Leftrightarrow x_{g,n}^{\text{cwo}}(t_{g}^{cwo})=k_{g}x_{C/C,n}^{\text{cwo}}(t_{g}^{cwo}),\text{ }k_{g}=\left|\frac{\lambda_{C/C,g}}{d_{g}}\right|.$$

Therefore,

$$V_{g,n}^{CWO}=x_{g,n}^{CWO}(t_{g})-x_{g,n-1}^{CWO}(t_{g})=k_{g}(x_{C/C,n}^{CWO}(t_{g})-x_{C/C,n-1}^{CWO}(t_{g})=k_{g}V_{C/C,n}^{CWO},\text{ }$$

hence,

$V_{g,n}^{CWO}=k_{g}V_{C/C,n}^{CWO},\text{ }k_{g}=\left|\frac{\lambda_{C/C,g}}{d_{g}}\right|.$ **(2.2)**

The values of $k_{g}=\left|\frac{\lambda_{C/C,g}}{d_{g}}\right|$ are 1.134, 1.24, 0.25, 0.9, and 0.384 for *per*, *tim*, *cwo*, *pdp1*, and *vri*, respectively. Furthermore, in the wt model,

$$\lambda_{C/C,g}x_{C/C,n}(t_{g})-\left|\lambda_{CWO,g}\right|x_{CWO,n}(t_{g})-\left|d_{g}\right|x_{g,n}(t_{g})=0\Leftrightarrow$$

$$x_{g,n}(t_{g})=k_{g}x_{C/C,n}(t_{g})-\frac{\left|\lambda_{CWO,g}\right|}{\left|d_{g}\right|}x_{CWO,n}(t_{g}),\text{ }k_{g}=\left|\frac{\lambda_{C/C,g}}{d_{g}}\right|$$

Therefore,

$V_{g,n}(t_{g})=x_{g,n}(t_{g})-x_{g,n-1}(t_{g})=k_{g}(x_{C/C,n}(t_{g})-x_{C/C,n}(t_{g})-\frac{\left|\lambda_{CWO,g}\right|}{\left|d_{g}\right|}(x_{CWO,n}(t_{g})-x_{g,n}(t_{g}),$ or

$$V_{g,n}(t_{g})=k_{g}V_{C/C,n}(t_{g})-\frac{\left|\lambda_{CWO,g}\right|}{\left|d_{g}\right|}V_{CWO,n}(t_{g}),\text{ }k_{g}=\left|\frac{\lambda_{C/C,g}}{d_{g}}\right|.$$

Consider the linear correlations $V_{CWO,n}(t_{g})\cong\alpha_{g}V_{g,n}(t_{g}),\text{ }\alpha_{g}>0$(see Figure 1d), thus

$V_{g,n}(t_{g})\cong\beta_{g}k_{g}V_{C/C,n}(t_{g}),\text{ }k_{g}=\left|\frac{\lambda_{C/C,g}}{d_{g}}\right|,\text{ }\beta_{g}=\frac{\left|d_{g}\right|}{\left|d_{g}\right|+\alpha_{g}\left|\lambda_{CWO,g}\right|}<1.$ **(2.3)**

The values of$\beta_{g}$ are 0.7394, 0.6640, 0.1096, 0.8741, 0.8929 at the peaks of *per*, *tim*, *cwo*, *pdp1*, and *vri*, respectively. The values of $\beta_{g}$ are 0.5181, 0.4651, 0.0058, 0.4533, 0.0943 at the trough levels of *per*, *tim*, *cwo*, *pdp1*, and *vri*, respectively.

**Linear correlations**

The linear equations (see Figure 1d) that correlate $V_{CWO,n}$and $V_{g,n}$ are as follows,

- At the peak time of *per*:

$y=0.15x-1.5*10^{-12},$ Norm of residuals = $1.05*10^{-6}$.

- At the peak time of *tim*:

$y=0.23x-2.3*10^{-12},$ Norm of residuals = $1.5*10^{-6}$.

- At the peak time of *cwo*:

$y=2.9x+2.3\text{*}10^{-12},$ Norm of residuals = $3.2*10^{-6}$.

- At the peak time of *pdp1*:

$y=0.18x-3.3\text{*}10^{-13},$ Norm of residuals = $6.3*10^{-7}$.

- At the peak time of *vri*:

$y=0.6x-2.8\text{*}10^{-12},$ Norm of residuals = $1.5*10^{-6}$.

- At the trough time of *per*:

$y=0.062x+2.4\text{*}10^{-12},$ Norm of residuals = $1.25*10^{-6}$.

- At the trough time of *tim*:

$y=0.05x+2.5\text{*}10^{-12},$ Norm of residuals = $1.26*10^{-6}$.

- At the trough time of *cwo*:

$y=0.59x+1.9\text{*}10^{-12},$ Norm of residuals = $1.01*10^{-6}$.

- At the trough time of *pdp1*:

$y=0.067x+2.5\text{*}10^{-12},$ Norm of residuals = $1.26*10^{-6}$.

- At the trough time of *vri*:

$y=0.16x+2.5\text{*}10^{-12},$ Norm of residuals = $1.27*10^{-6}$.

**Computing the spectrum of the LE by the discrete QR algorithm**

I consider the computation of the full spectrum of LE for a continuous finite dimensional dynamical system by the discrete QR method. Let be continuous dynamical system defined by a diffeormorphic flow map acting on a 15-dimensional space M:

**(3.1)**

The continuous dynamical system is given by a set of ordinary differential equations,

**(3.2)**

where $v$ is continuously differentiable. Here the overdot denotes differentiation with respect to $t$, which will be called time. The 15-dimensional space M will be called the phase space. This set of equations has an oscillatory numerical solution, $y_{c}(t),$called the central orbit. The computation of the Lyapunov exponent is based on the linearized flow map,

**(3.2)**

With respect to the orthonormal standard basis $\left\{e_{1},...,e_{15}\right\}$in the tangent spaces $T_{x}M$and . The linearized flow map is given as the invertible flow matrix $Y=Y(x,t).$ Linearizing eq. (1.2) yields

$\delta\mathop{\mathop{y_{i}}\limits^{\bullet} =J_{ij}}\limits[y_{c}(t)]\delta y_{j},$ **(3.4)**

where the 15X15 matrix $J_{ij}$ is given by

$J_{ij}=\left.\frac{\partial v_{i}}{\partial y_{j}}\right|_{y=y_{c}(t)}$ **(3.5)**

Here $J$ is the Jacobi matrix of the partial derivatives of the vector field $v$ at the point $y_{c}(t).$ We introduce the time displacement operator that takes an initial vector at $t=t_{0}$ to a final vector at $t=t_{end}.$ Thus

$Y(t_{end},t_{0})=\frac{\delta y(t_{end})}{\delta y(t_{0})}$.

It follows that the tangent map Y satisfies the differential equation:

$\mathop{\mathop{Y}\limits^{\bullet} =JY.}\limits$ **(3.6)**

Small perturbations to the orbits $x(t)$ evolve according to the dynamics of the linear variational equation

$\mathop{Y}\limits^{\bullet} =JY,Y(x,0)=I.$ **(3.7)**

The Lyapunov exponents $\lambda_{i}$ are given by the logarithms of the eigenvalues $\mu_{i}$, $i=1,...,15,$ of the positive and symmetric matrix,

$\Lambda=\lim_{t\to\infty} (Y(x,t)^{T}Y(x,t))^{\frac{1}{2t}},$ **(3.8)**

Where $Y(x,t)^{T}$denotes the transpose of $Y(x,t).$ The existence of $\Lambda$is based on the multiplicative ergodic theorem proved by Oseledec [16]. The Lyapunov exponents describe the way nearby trajectories converge or diverge in the state space by measuring the mean logarithmic growth rates. The Lyapunov exponents are denotes by $\lambda_{1}\geq\lambda_{2}\geq...\geq\lambda_{15}$. The theorem by Oseledec leads to an equivalent way to characterize the LEs (see also [17–19]). Let $E^{(i)}$ be the subspace of corresponding to the eigenvalues of $\Lambda$in (1.8) whose logarithms are less than $\lambda_{i}$, so that Let $p_{k}\in E^{(k)}/E^{(k+1)},$ then one has

$\lambda_{k}=\lim_{t\to\infty} \frac{1}{t}\log\left\|Y(t)p_{k}\right\|,$ **(3.9)**

where is the 2-norm. More details on the meaning of the Lyapunov exponents can be found in the following articles [17–21].

We use the discrete QR algorithm to approximate the LE [22–24]. Let The following equations are integrated numerically from $t=i$ to $t=i+1$:

$\left\{\begin{matrix} \frac{dy}{dt}=v(t,y),\text{ }y(i)=y^{i},y(0)=I; \\ \frac{dZ}{dt}=v_{y}(t,y)Z(t)=J(t)Z(t),\text{ }Z(i)=Q_{i},\text{ }Q_{0}=I, \end{matrix}\right.$ **(3.10)**

Where $y^{i}$ and $Z^{i}$ denote the values of $y(t)$ and $Z(t)$ computed numerically from $t=i-1$ to $t=i,$ respectively. Then the *computed* $Z^{i+1}$ is decomposed as $Q_{i+1}R_{i+1},$ where $R_{i+1}$ is upper triangular with positive diagonal entries and $Q_{i+1}$ is orthonormal. Observe that the orthonormal basis changes at each step. We obtain the LEs as:

$\lambda_{k}=\lim_{i\to\infty} \frac{1}{i}\log((R_{i})_{kk}...(R_{1})_{kk})=\lim\frac{1}{i}_{i\to\infty} \sum_{j=1}^{i} \log((R_{j})_{kk}),1\leq k\leq15.$ **(3.11)**

The LE spectrum of the wt and mutant *cwo*-models in LD and DD are shown in Figure 3a and Figure S5.

**The Lorenz equation**

To illustrate the application of the QR method to a well known system, we turn to the standard case of the Lorenz equations:

$\begin{matrix} \mathop{z}\limits^{\bullet} _{1}=\sigma(z_{2}-z_{1}), \\ \mathop{z}\limits^{\bullet} _{2}=z_{1}(\rho-z_{3})-z_{2}, \\ \mathop{z}\limits^{\bullet} _{3}=z_{1}z_{2}-\beta z_{3} \end{matrix}$ **(3.12)**

We used parameter values $\sigma=10,\text{ }\rho\text{= 28,}$ and $\beta=8/3.$ Our results (Figure S5) are consistent with other reports [25,26]. The LE converge at $0.9055$, $-4.9865x10^{-7}$, and $-14.5721$; their sum is -13.6667 = $-\sigma-1-\beta,$ as expected from equation (3.12).

**Reference List**

1. Glossop NR, Lyons LC, Hardin PE. (1999) Interlocked feedback loops within the Drosophila circadian oscillator. Science 286: 766-768.

2. Glossop NR, Houl JH, Zheng H, Ng FS, Dudek SM, et al. (2003) VRILLE feeds back to control circadian transcription of Clock in the Drosophila circadian oscillator. Neuron 37: 249-261.

3. Blau J, Young MW (1999) Cycling vrille expression is required for a functional Drosophila clock. Cell 99: 661-671.

4. McDonald MJ, Rosbash M (2001) Microarray analysis and organization of circadian gene expression in Drosophila. Cell 107: 567-578.

5. Xie Z, Kulasiri D (2007) Modelling of circadian rhythms in Drosophila incorporating the interlocked PER/TIM and VRI/PDP1 feedback loops. J Theor Biol 245: 290-304.

6. Smolen P, Hardin PE, Lo BS, Baxter DA, Byrne JH (2004) Simulation of Drosophila circadian oscillations, mutations, and light responses by a model with VRI, PDP-1, and CLK. Biophys J 86: 2786-2802.

7. Cyran SA, Buchsbaum AM, Reddy KL, Lin MC, Glossop NR, et al. (2003) vrille, Pdp1, and dClock form a second feedback loop in the Drosophila circadian clock. Cell 112: 329-341.

8. Gekakis N, Saez L, Delahaye-Brown AM, Myers MP, Sehgal A, et al. (1995) Isolation of timeless by PER protein interaction: defective interaction between timeless protein and long-period mutant PERL. Science 270: 811-815.

9. Saez L, Young MW (1996) Regulation of nuclear entry of the Drosophila clock proteins period and timeless. Neuron 17: 911-920.

10. Marrus SB, Zeng H, Rosbash M (1996) Effect of constant light and circadian entrainment of perS flies: evidence for light-mediated delay of the negative feedback loop in Drosophila. EMBO 15: 6877-6886.

11. Lee C, Bae K, Edery I (1999) PER and TIM inhibit the DNA binding activity of a Drosophila CLOCK-CYC/dBMAL1 heterodimer without disrupting formation of the heterodimer: a basis for circadian transcription. Mol Cell Biol 19: 5316-5325.

12. Kloss B, Price JL, Saez L, Blau J, Rothenfluh A, Wesley CS, et al. (1998) The Drosophila clock gene double-time encodes a protein closely related to human casein kinase Iepsilon. Cell 94: 97-107.

13. Price JL, Blau J, Rothenfluh A, Abodeely M, Kloss B, et al. (1998) double-time is a novel Drosophila clock gene that regulates PERIOD protein accumulation. Cell 94: 83-95.

14. Yu W, Zheng H, Houl JH, Dauwalder B, Hardin PE (2006) PER-dependent rhythms in CLK phosphorylation and E-box binding regulate circadian transcription. Genes Dev 20: 723-733.

15. Fathallah-Shaykh H.M., Bona J.L., Kadener S. (2009) Mathematical model of the Drosophila circadian clock: loop regulation and transcriptional integration. Biophys J 97: 2399-2408.

16. Oseledec V I (2008) A multiplicative ergodic theorem. Characteristic Ljapunov, exponents of dynamical systems. Trans Moscow Math Soc 19: 197-231.

17. Benettin G, Galgani L, Giorgilli A, Strelcyn J-M (1980) Lyapunov exponents for smooth dynamical systems and for hamiltonian ststems; a moethod for computing all of them. Part I: theory. Meccanica 15: 9-20.

18. Benettin G, Galgani L, Giorgilli A, Strelcyn J-M (1980) Lyapunov exponents for smooth dynamical systems and for hamiltonian ststems; a moethod for computing all of them. Part II: Numerical Applications. Meccanica 15: 21-30.

19. Eckmann J-P, Ruelle D (1985) Erogodic theory of chaos and strange attractors. Rev Modern Phys 57: 617-656.

20. Goldhirsch I, Sulem P-L (1987) stability and Lyapunov stability of dynamical systems: a differential approah and a numerical method. Physica D 27: 311-337.

21. Greene J M, Kim J-S (1987) The calculation of Lyapunov spectra. Physica D 24: 213-225.

22. Geist K, Ulrich P, Lauterborn W (1990) Comparison of different methods for computing Lyapunov exponents. Prog Theor Phys 83: 875-893.

23. Dicci L, Van Vleck E S (1995) Computation of a few Lyapunov exponents for continuous and discrete dynamical systems. Appl Numer Math 17: 275-291.

24. Dieci L, Russell R D, Van Vleck E S (1997) On the computation of Lyapunov exponents for contnuous dynamical systems. SIAM J Numer Anal 34: 402-423.

25. Christiansen F, High H H (1997) Computing Lyapunov spectra with continuous Gram-Schmidt Orthonormalization. Nonlinearity 10: 1063-1072.

26. Rangarajan G, Habib S, Ryne R D (1998) Lyapunov exponents without rescaling and reorthogonalization. Phys Rev Lett 80: 3747-3750.
