# Supplementary figures and images for: Dynamics of the Drosophila Circadian Clock: Theoretical Anti-Jitter Network and Controlled Chaos
Source: PLoS One. 2010 Oct 13;5(10):e11207. doi: 10.1371/journal.pone.0011207 (PMC2954144; doi:10.1371/journal.pone.0011207)

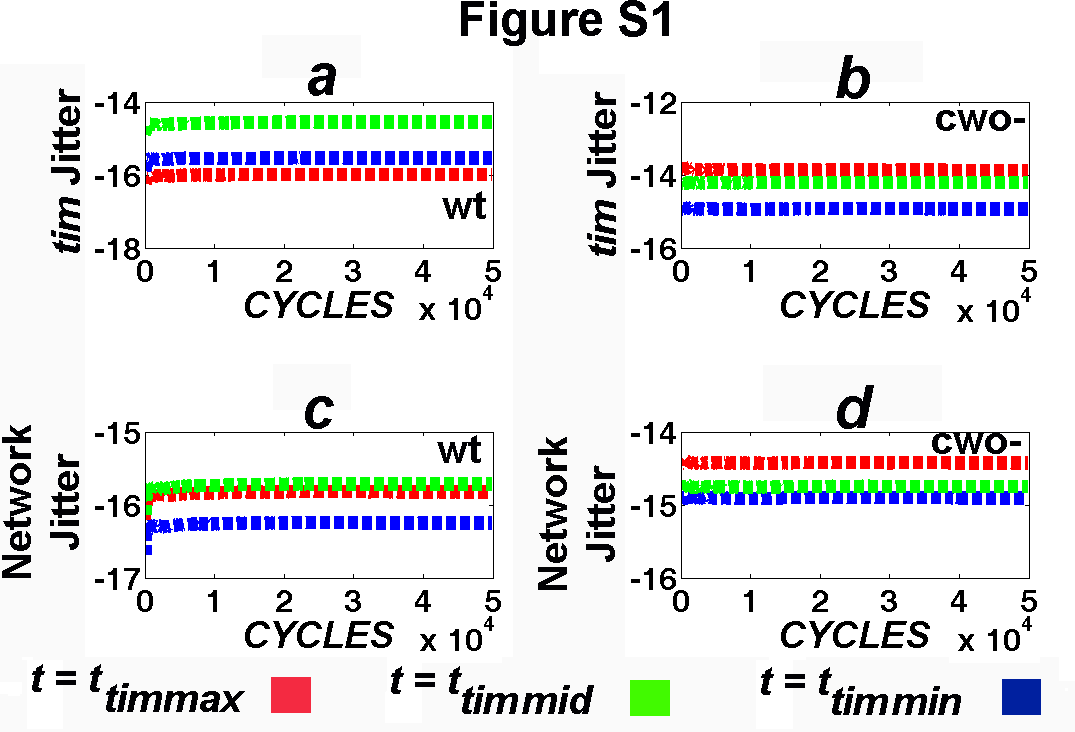

Supplement: Figure S1 — CWO lowers tim and network jitters. Shown are the tim and network jitters of the wt (a,c) and cwo-mutant (b,d) models in LD at t ∈ {ttimmin, ttimmid, ttimmax} starting from cycle 100. (2.37 MB TIF) [file pone.0011207.s002.tif]

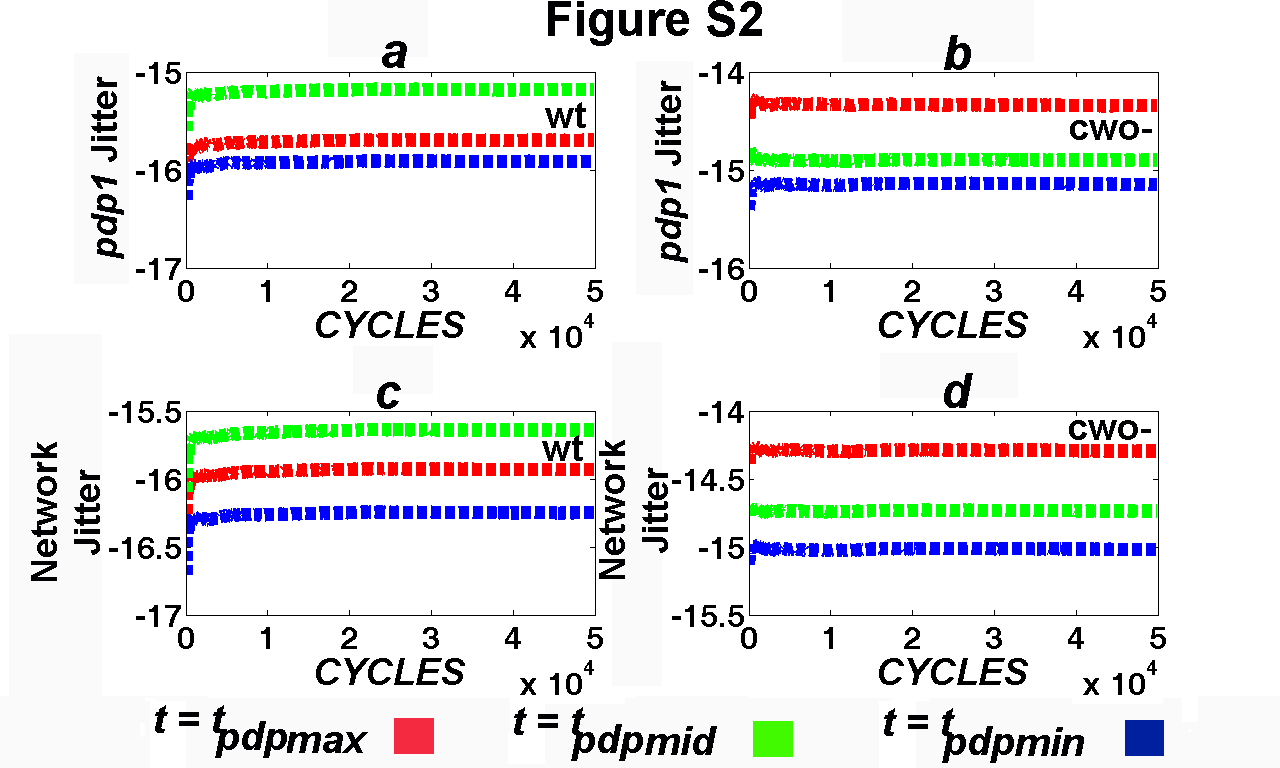

Supplement: Figure S2 — CWO lowers Pdp1 and network jitters. Shown are the Pdp1 and network jitters of the wt (a,c) and cwo-mutant (b,d) models in LD at t ∈ {tpdpmin, tpdpmid, tpdpmax} starting from cycle 100. (2.96 MB TIF) [file pone.0011207.s003.tif]

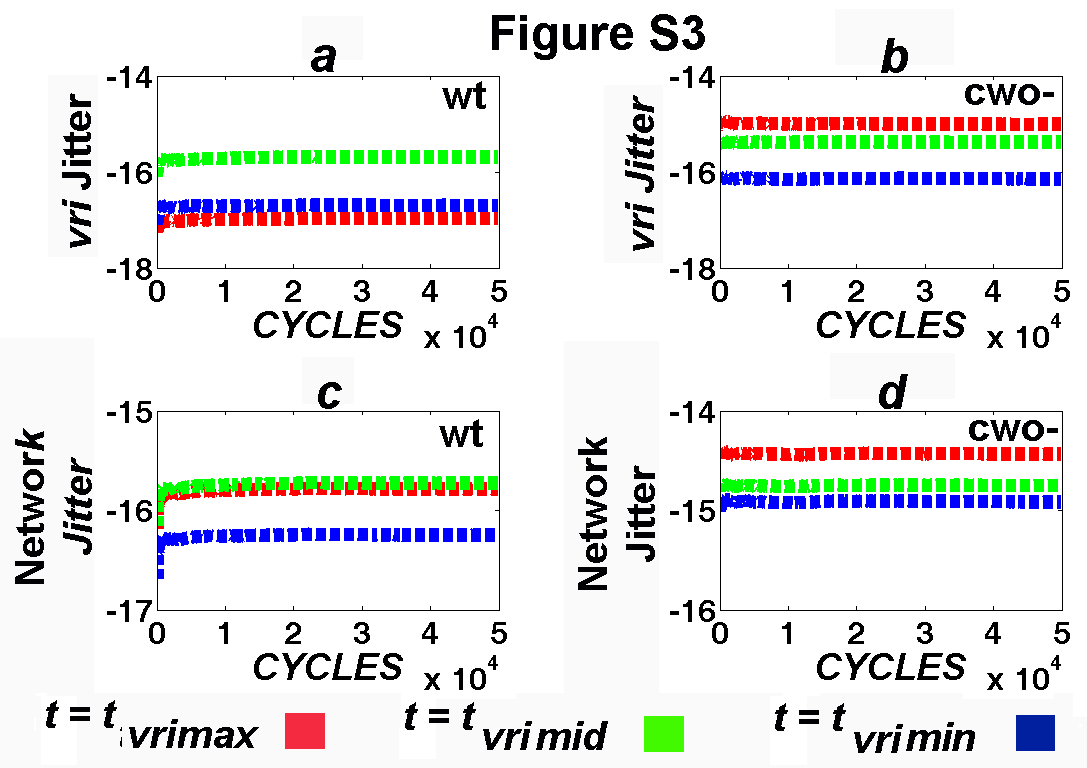

Supplement: Figure S3 — CWO lowers vri and network jitters. Shown are the vri and network jitters of the wt (a,c) and cwo-mutant (b,d) models in LD at t ∈ {tvrimin, tvrimid, tvrimax} starting from cycle 100. (2.52 MB DOC) [file pone.0011207.s004.tif]

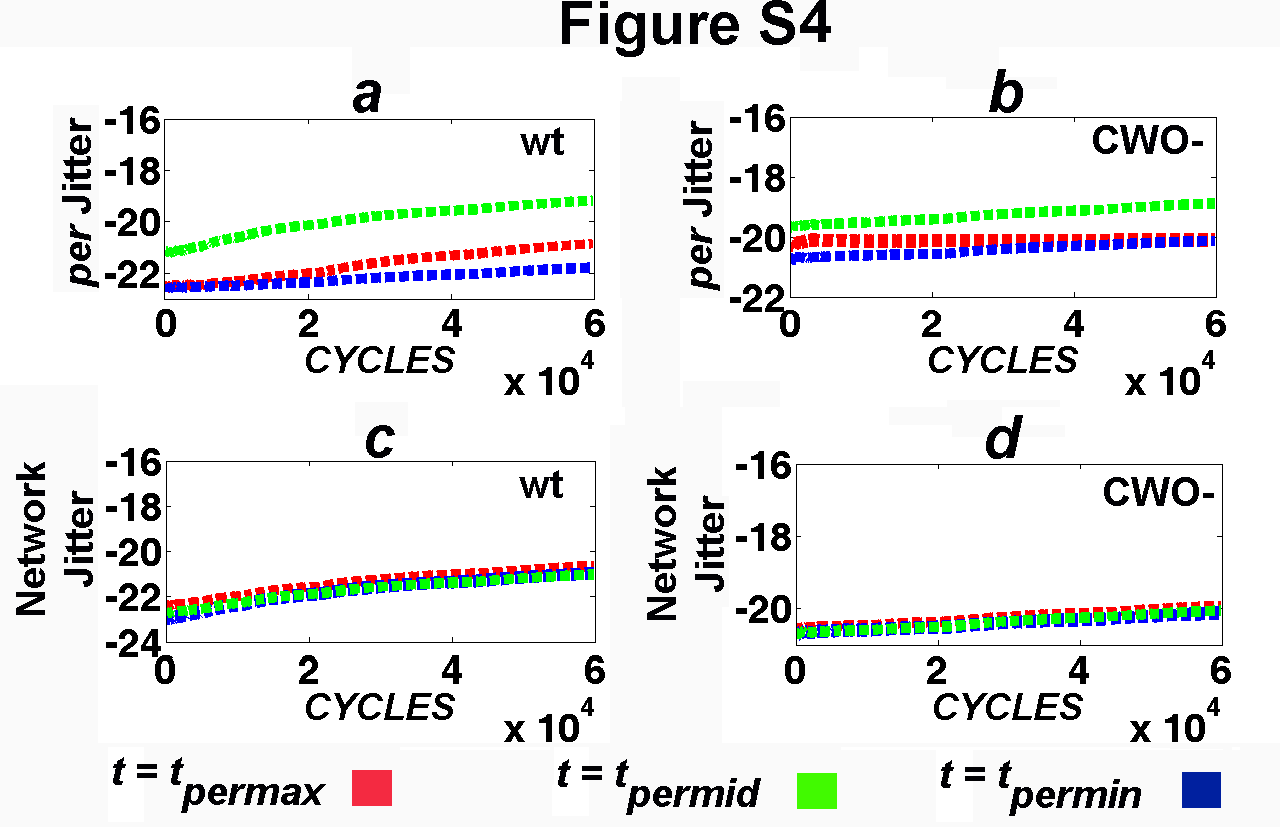

Supplement: Figure S4 — CWO lowers direct target and network jitters, second numerical method. These results are computed by ode15s (see Figure 2 legend); shown are the plots of per and network jitters of the wt (a,c) and cwo-mutant (b,d) models in LD at t ∈ {tpermin, tpermid, tpermax} starting from cycle 100. (3.19 MB TIF) [file pone.0011207.s005.tif]

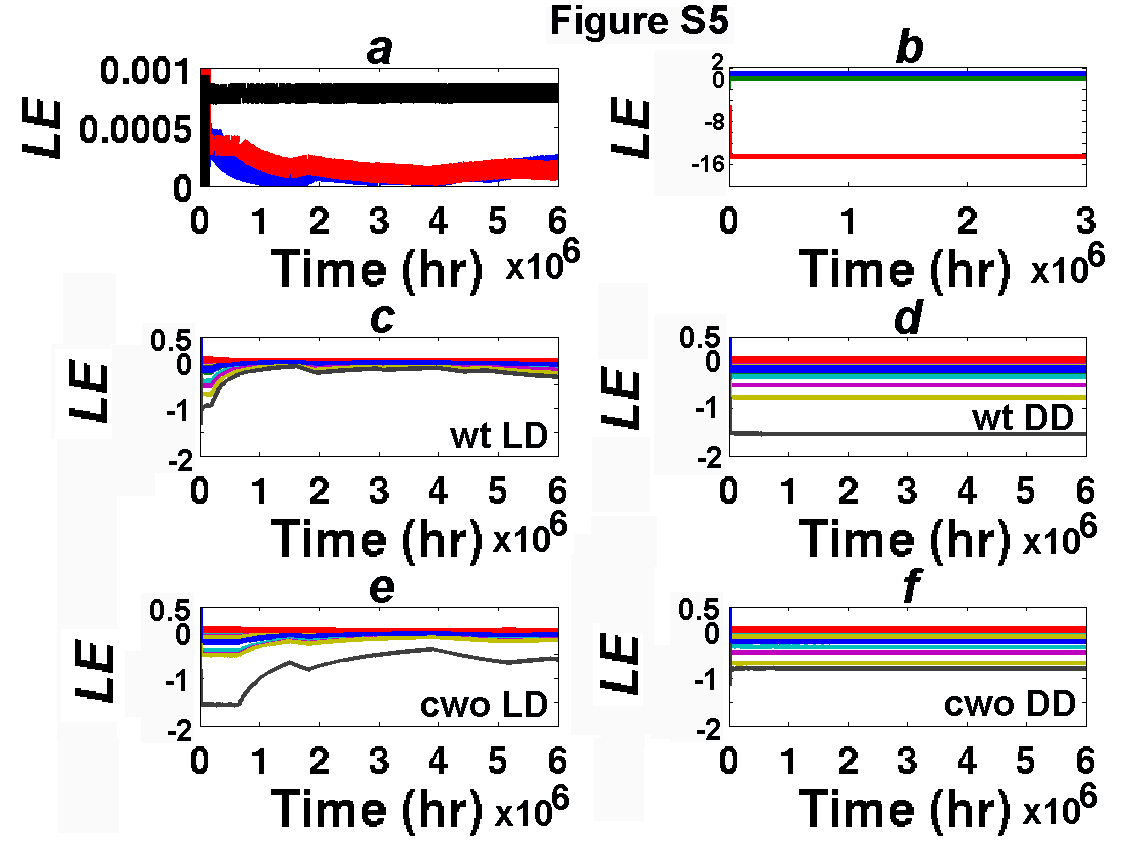

Supplement: Figure S5 — The Lyapunov characteristic exponents. (a) plots the second positive LE of the wt model in LD (blue), wt model in DD (black) and the cwo-mutant model in LD (red). (b) plots the LE for the Lorenz equations (σ = 10, ρ = 28 and β = 8/3). (c–f) plot the full LE spectrum of the wt and cwo-mutant models in LD and DD conditions. (2.87 MB TIF) [file pone.0011207.s006.tif]

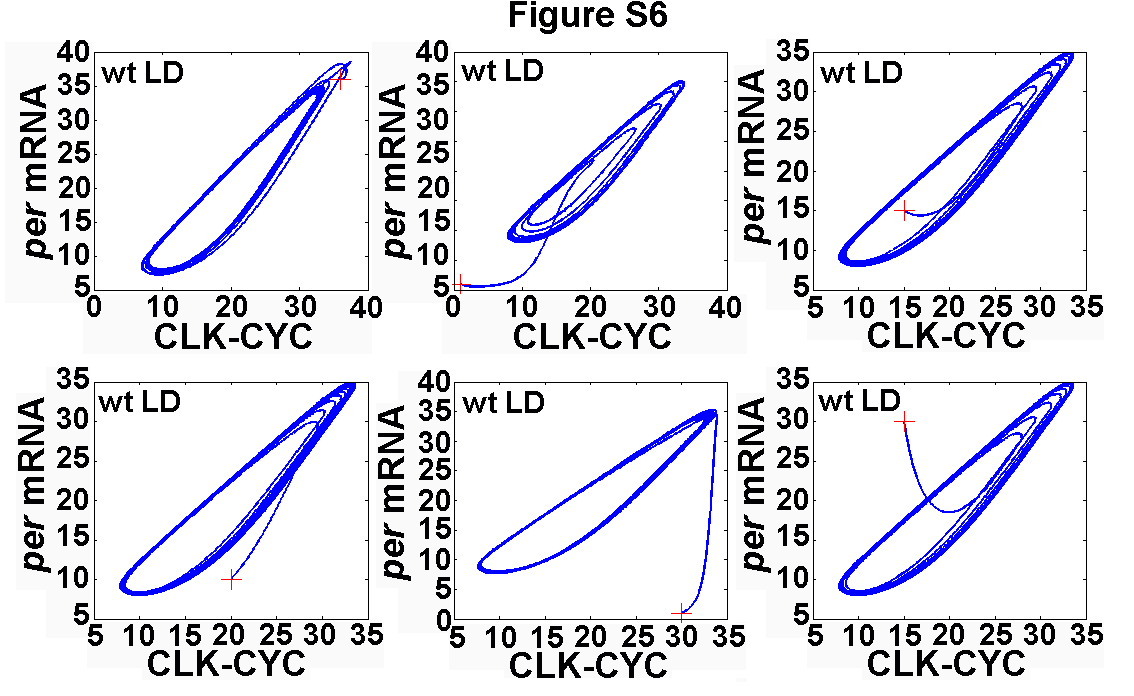

Supplement: Figure S6 — Attractor to stable limit cycle; wt model in LD. Shown are the trajectories of the wt model in LD starting from different points in the phase space (red X) and converging to a stable limit cycle (cycles 1–120000). (2.38 MB TIF) [file pone.0011207.s007.tif]

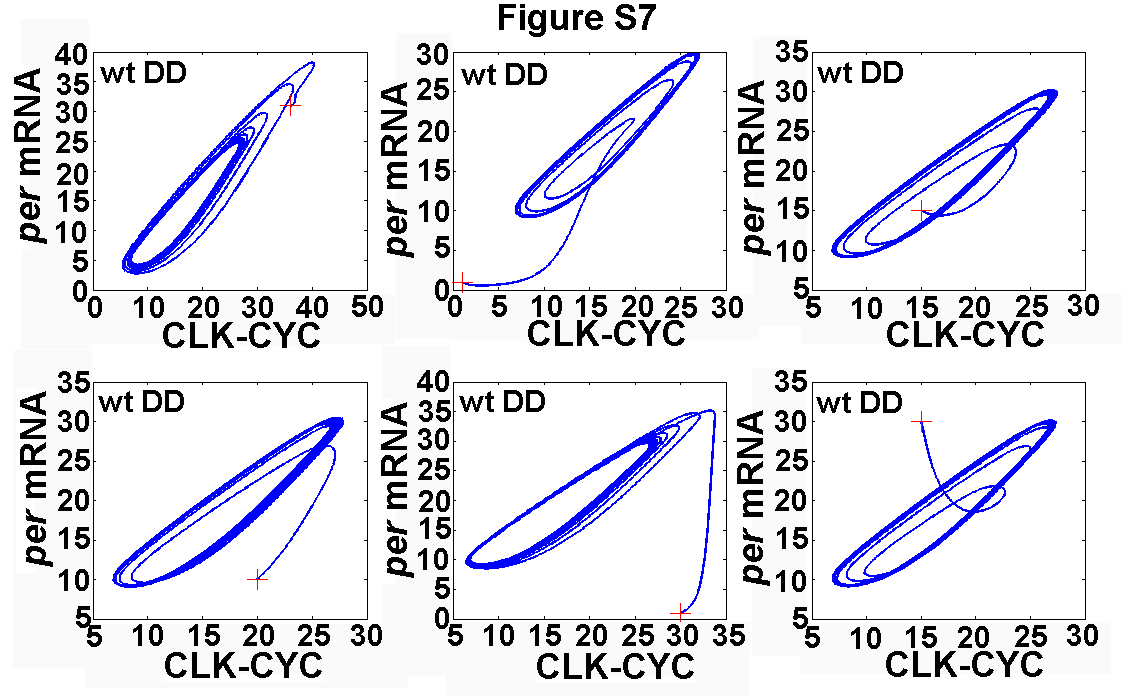

Supplement: Figure S7 — Attractor to stable limit cycle; wt model in DD. Shown are the trajectories of the wt model in DD starting from different points in the phase space (red X) and converging to a stable limit cycle (cycles 1–120000). (2.38 MB TIF) [file pone.0011207.s008.tif]

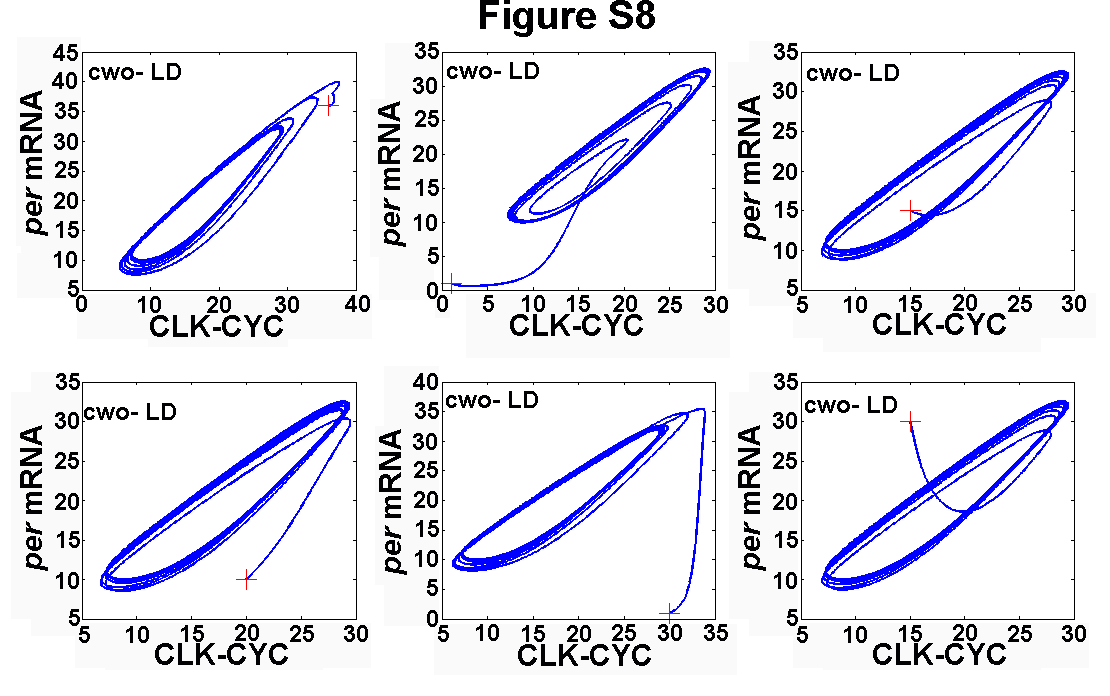

Supplement: Figure S8 — Attractor to stable limit cycle; cwo-mutant model in LD. Shown are the trajectories of the cwo-mutant model in LD starting from different points in the phase space (red X) and converging to a stable limit cycle (cycles 1–120000). (2.33 MB TIF) [file pone.0011207.s009.tif]

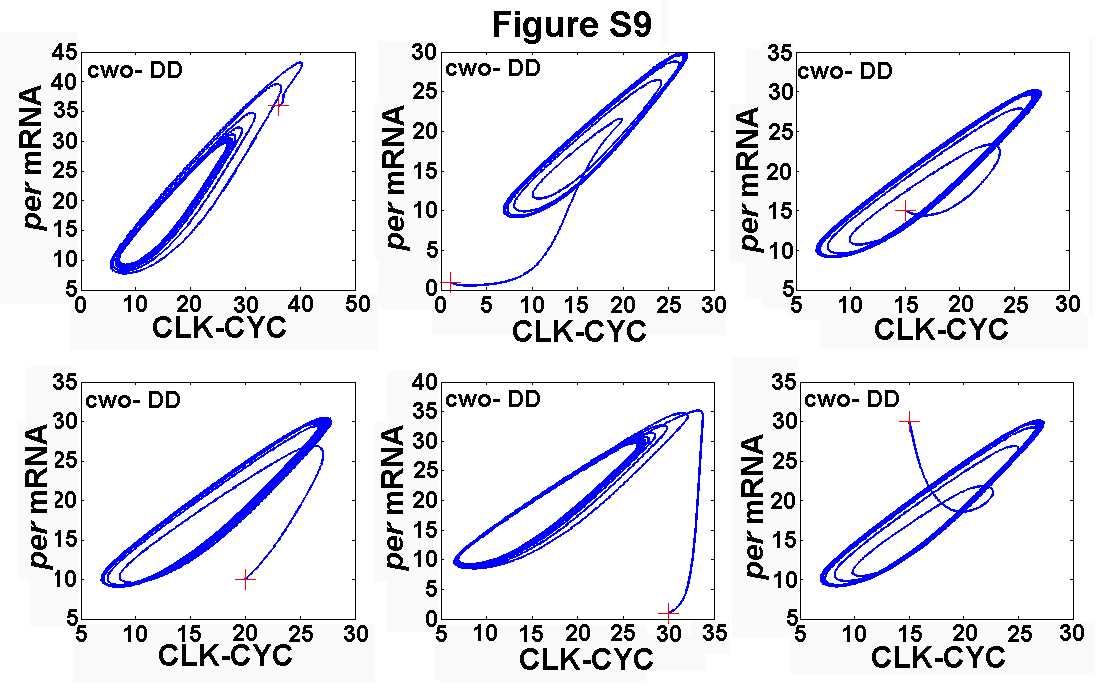

Supplement: Figure S9 — Attractor to stable limit cycle, cwo-mutant model in DD. Shown are the trajectories of the cwo-mutant model in DD starting from different points in the phase space (red X) and converging to a stable limit cycle (cycles 1–120000). (2.33 MB TIF) [file pone.0011207.s010.tif]

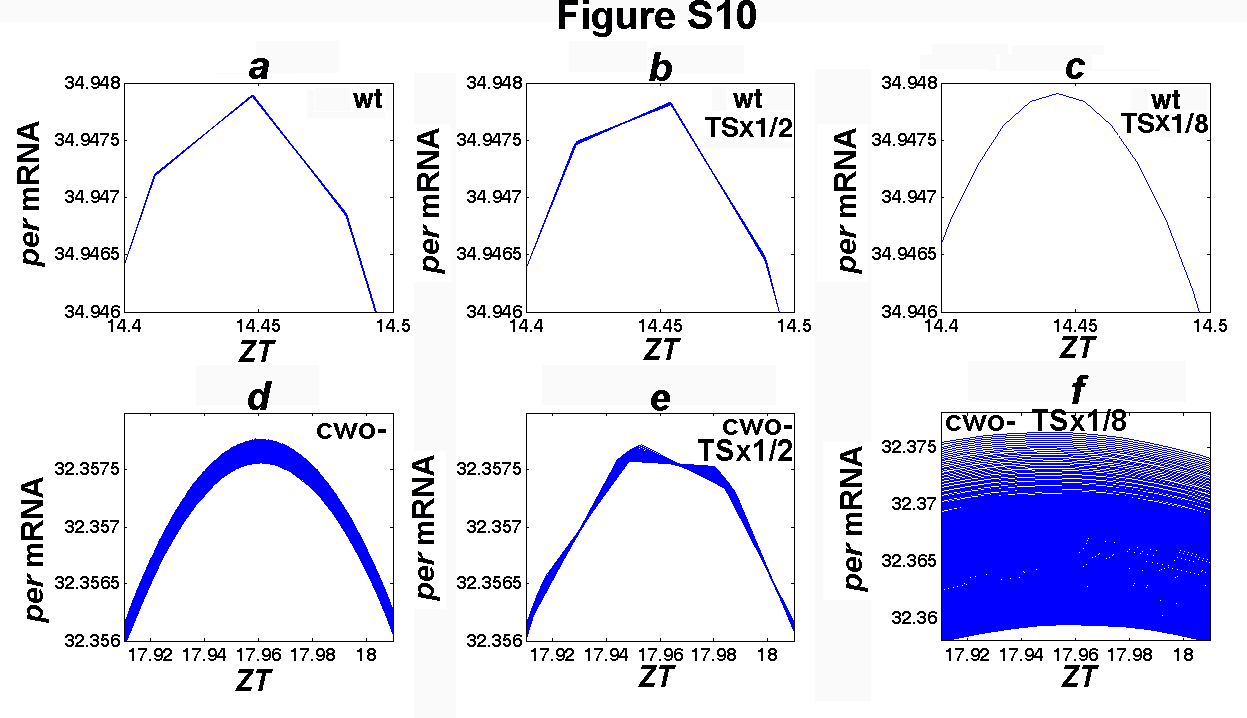

Supplement: Figure S10 — Jitters persist after lowering maximal time steps. Shown are the variations in per mRNA oscillations of the wt (a–c) and cwo-mutant (d–f) models (cycles 100–1500) when the maximal time step of ode45 is not changed (a and d), multiplied by 1/2 (b and e) and 1/8 (c and f). (2.70 MB TIF) [file pone.0011207.s011.tif]
